# Draft Genome Sequence of the Novel *Peptoniphilus* sp. Strain ChDC B134, Isolated from a Human Periapical Abscess Lesion

**DOI:** 10.1128/genomeA.00822-13

**Published:** 2013-10-24

**Authors:** Eugene Cho, Soon-Nang Park, Hak Kyun Kim, Dae-Soo Kim, Jaeeun Jung, Jeong-Hun Baek, Yun Kyong Lim, Eojin Jo, Mi-Hwa Choi, Young-Hyo Chang, Yeseul Shin, Jayoung Paek, Jeong Hwan Shin, Jihun Kim, Sang-Haeng Choi, Hong-Seog Park, Hongik Kim, Joong-Ki Kook

**Affiliations:** Korean Collection for Oral Microbiology and Department of Oral Biochemistry, School of Dentistry, Chosun University, Gwangju, Republic of Koreaa; Department of Oral and Maxillofacial Surgery, School of Medicine, Chung-Nam National University, Daejeon, Republic of Koreab; Human Derived Material Center, Korea Research Institute of Bioscience and Biotechnology (KRIBB), Daejeon, Republic of Koreac; Macrogen Inc., Gasan-dong, Seoul, Republic of Koread; Korean Collection for Type Cultures, Biological Resource Center, KRIBB, Daejeon, Republic of Koreae; Department of Laboratory Medicine, Busan Paik Hospital, Inje University College of Medicine, Busan, Republic of Koreaf; Research Institute of Unitech Science Co., Ltd., Daejeon, Republic of Koreag; R&D Division, Vitabio Inc., Daejeon, Republic of Koreah

## Abstract

The genus *Peptoniphilus* comprises butyrate-producing, nonsaccharolytic species that use peptone and amino acids as major energy sources. The novel *Peptoniphilus* sp. strain ChDC B134 (=KCOM 1628) was isolated from a human periapical abscess lesion. Here, we report the draft genome sequence of the strain.

## GENOME ANNOUNCEMENT

*Peptoniphilus* species are Gram-positive anaerobic cocci and are butyrate-producing, nonsaccharolytic species that use peptone and amino acids as major energy sources (1–4). The novel *Peptoniphilus* sp. strain ChDC B134 (=KCOM 1628) was isolated from a human periapical abscess lesion. In this report, we present the draft genome sequence of *Peptoniphilus* sp. ChDC B134.

Draft sequencing was performed by Macrogen Co. (Seoul, South Korea) using the Illumina HiSeq 2000 system sequencing technology. We constructed 101 paired-end sequencing libraries with insert sizes of about 200 bp and generated 39,093,740 bp of usable sequence. We assembled the reads using SOAP*denovo* (http://soap.genomics.org.cn). SOAP*denovo* version 1.05 was run with option K79 and the configuration options of reverse_seq of 0 (standard mate-pair orientation), asm_flags of 3 (try harder to build large contigs), and rank of 1 (reads were used while scaffolding). The reads were assembled into five contigs with a size range of 44,641 to 829,742 bp (total 1,499,854 bp), and the percentage of G+C content is 35.5%. Open reading frames were predicted and annotated using the Glimmer 3.02 modeling software package (5). The predicted protein sequences were annotated in the Gene Ontology (GO) database using the Basic Local Alignment Search Tool (BLAST). Next, the GO classes were grouped into a total of 124 GO-Slim terms using the web tool CateGOrizer (6).

The draft genome sequence contains 1,471 protein-coding genes, four copies of 16S rRNA, 47 tRNA genes, and several key pathways for amino acids, carbohydrates, lipids, and organic acids. Biosynthetic pathways exist for at least 6 amino acids, including asparagine, aspartate, threonine, proline, glutamate, and glutamine. The draft genome sequence encodes virulence factors, such as a vancomycin B-type resistance protein, a multiantimicrobial extrusion protein, a macrolide export ATP-binding/permease protein, a multidrug and toxin extrusion family efflux pump, hemolysin, programmed cell death toxin (YdcE), a metallo-β-lactamase family protein, a butyrate fermentation-related protein, and membrane-associated zinc metalloprotease. The sequence contains sporulation-related genes, such as those for sporulation protein (SpoIID) and sporulation initiation inhibitor protein (Soj). The genome contains oxidative stress response genes, such as genes for iron-binding ferritin-like antioxidant protein, superoxide reductase, ferroxidase, rubrerythrin, and glutaredoxin, but not for glutathione peroxidase. The sequence also encodes four sensor kinases and five response regulators of two-component systems.

### Nucleotide sequence accession numbers.

This whole-genome shotgun project has been deposited at DDBJ/EMBL/GenBank under the accession no. AUQY00000000. The version described in this paper is the first version, AUQY01000000.
